# Classification of thyroid nodules using ultrasound images

**DOI:** 10.6026/97320630016145

**Published:** 2020-02-29

**Authors:** T Manivannan, Nagarajan Ayyappan

**Affiliations:** 1 Department of Computer Applications, Alagappa University, Karaikudi, Tamilnadu, India

**Keywords:** Ultrasonography, thyroid nodules, Computer Aided Diagnosis (CAD), feature extraction

## Abstract

Medical imaging using image sensors play an essential role in effective diagnosis. Therefore, it is of interest to use medical imaging techniques for the diagnosis of thyroid-linked dysfunction. Ultrasound is the low-cost image processing technique to study internal organs and blood flow in blood vessels. Digital processed images help to distinguish between normal, benign and malignant tissue stages in organs.Hence, it is of importance to discuss the design and development of a computer-aided image-processing model for thyroid nodule identification, classification and diagnosis.

## Background

Medical image management in Hospital information system (HIS) is gaining momentum in recent years [1].The use of image processing techniques and tools in the early diagnosis of diseases has become routine in modern healthcare [1].Various imaging technologies such as Photo acoustic imaging, Radiology, Magnetic Resonance Imaging (MRI), Tomography and Ultrasound imaging are available. Early diagnosis facilitates treatment. Ultrasound images help to differentiate benign and malignant lesions using image-processing models [2].Computer Aided Diagnosis (CAD) minimizes errors created due to subjective interpretation and assists to make a fast accurate diagnosis. Moreover, a computer-aided diagnosis (CAD) system can be helpful to cross-verify the severity of nodules. Therefore, it is of interest to use medical imaging techniques for the diagnosis of thyroid-linked dysfunction [3].Simple diagnostic methods such as (a) physical examination know the general body conditions of the patient and (b) Continuous temperature updates for measuring thyroid functions of the patients [4] are available.Pathological diagnostic methods such as (a) thyroid function test [6] and (b) radio immunoassay based detection method based upon patient blood samples are known. Diagnosis by imaging methods such as the ultra sonography transducers ultra sonography produce high frequency signals when administered to the neck where the transducer gathers the sound waves to create an image [5].It clearly shows the entire structure of the body and movement of the internal organs to help locate the thyroid glands in exact shape and size. Other methods such as Positron Emission Tomography (PET scan) which detects the gamma rays with the help of tracer and they are displayed on monitor [7] are also available. The X-ray images recorded on a film called radiograph [8] is useful in this context. It produced the images based on absorption rates. So the tissues are displayed in light or dark. CT Scan used computerized tomography, which makes the combinations of X-rays images and finally it produces cross tomography is also known for this purpose.

## Materials and Methods:

### Image preprocessing:

The medical imaging requires significant quality of digital images for perfect and efficient diagnosis. Medical images have noise due to some unwanted signals during transmission [9].Hence, denoising process is compulsory to obtain a high quality medical image. Image denoising is a process of removing the noise. Denoising process is used to analyze and improve the image quality [10]. Various kinds of noises are present in an image. These include additive noises and multiplicative noises. The filtering techniques remove speckle noise from image. It is re-sized to have the same distance scale which is the physical space represented by each pixel in the image. This step is used to improve the quality of the image by removing the undesired distortion from the image. Dust particles in the image from source or heat can cause some noise [9].Clipping of the ultrasound image is performed to get region of interest.Image smoothing is done using some filtering techniques.Image enhancement is also done too increase contrast. Thus,image smoothing helps in increasing the detection of nodules accurately.

### Extraction of features:

The feature extraction is obtained by Segmentation-based fractal texture analysis (SFTA). It used to extract several independent features that decompose the image into set of binary values.

### Classification:

This stage classifies the detected nodule as malignant or benign by using the described classification model using adequate machine learning technique.

### Analysis of the model:

The described model provides the potential with improved accuracy in the detection of thyroid cancer using segmentation and classification techniques. The noise removal techniques remove the noises and hence false positive is reduced.

## Conclusions:

It is of interest to use medical imaging techniques for the diagnosis of thyroid-linked dysfunction. Ultrasound is the low-cost image processing technique to study internal organs and blood flow in blood vessels. The described CAD model uses independent features. We report the importance of the design and development of a computer-aided image-processing model for thyroid nodule identification, classification and diagnosis.

## Figures and Tables

**Figure 1:**
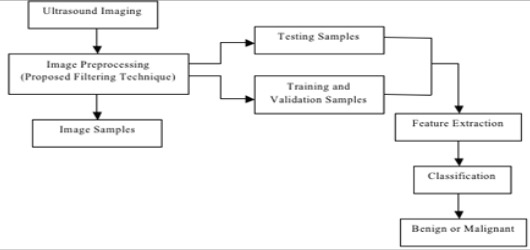
Flowchart for the classification of thyroid nodules using ultrasound images.
